# Succinyl-proteome profiling of *Dendrobium officinale*, an important traditional Chinese orchid herb, revealed involvement of succinylation in the glycolysis pathway

**DOI:** 10.1186/s12864-017-3978-x

**Published:** 2017-08-10

**Authors:** Shangguo Feng, Kaili Jiao, Hong Guo, Mengyi Jiang, Juan Hao, Huizhong Wang, Chenjia Shen

**Affiliations:** 1 College of Life and Environmental Sciences, Hangzhou Normal University, Hangzhou, 310036 China; 2 Zhejiang Provincial Key Laboratory for Genetic Improvement and Quality Control of Medicinal Plants, Hangzhou Normal University, Hangzhou, 310036 China

**Keywords:** *D. officinale*, Glycolysis pathway, LC-MS/MS, Metabolic pathway, Succinylation

## Abstract

**Background:**

Lysine succinylation is a ubiquitous and important protein post-translational modification in various eukaryotic and prokaryotic cells. However, its functions in *Dendrobium officinale*, an important traditional Chinese orchid herb with high polysaccharide contents, are largely unknown.

**Results:**

In our study, LC-MS/MS was used to identify the peptides that were enriched by immune-purification with a high-efficiency succinyl-lysine antibody. In total, 314 lysine succinylation sites in 207 proteins were identified. A gene ontology analysis showed that these proteins are associated with a wide range of cellular functions, from metabolic processes to stimuli responses. Moreover, two types of conserved succinylation motifs, ‘***K^suc^******K**’ and ‘****EK^suc^***’, were identified. Our data showed that lysine succinylation occurred on five key enzymes in the glycolysis pathway. The numbers of average succinylation sites on these five enzymes in plants were lower than those in bacteria and mammals. Interestingly, two active site amino acids residues, K103 and K225, could be succinylated in fructose-bisphosphate aldolase, indicating a potential function of lysine succinylation in the regulation of glycolytic enzyme activities. Furthermore, the protein–protein interaction network for the succinylated proteins showed that several functional terms, such as glycolysis, TCA cycle, oxidative phosphorylation and ribosome, are consisted.

**Conclusions:**

Our results provide the first comprehensive view of the succinylome of *D. officinale* and may accelerate future biological investigations of succinylation in the synthesis of polysaccharides, which are major active ingredients.

**Electronic supplementary material:**

The online version of this article (doi:10.1186/s12864-017-3978-x) contains supplementary material, which is available to authorized users.

## Background

Protein post-translational modification (PTM) is an efficient strategy for expanding the structural diversity of proteins and for increasing the regulation of cellular physiology [1, 2]. Several covalent modifications, including phosphorylation, ubiquitination, glycosylation, methylation and acetylation, have been well studied over the past years [3–6]. Lysine succinylation, a widespread reversible protein PTM, has recently been identified in both eukaryotic and prokaryotic species [7, 8]. In contrast to methylation and acetylation, succinylation results in a more substantial alteration in the properties, including stability and conformational space, of some lysine-containing proteins [9, 10]. However, the regulatory mechanism of succinylation is largely unknown.

The identification of succinylated proteins is the first step in revealing the roles of protein succinylation in various biological processes [11]. Since first being verified in three *Escherichia coli* proteins [9], succinylation profiles have subsequently been identified in animal tissues [12]. Recently, lysine succinylation has also been widely investigated in various organisms, including bacteria (*Vibrio parahemolyticus*, *Corynebacterium glutamicum* and *Mycobacterium tuberculosis*), fungi (*Saccharomyces cerevisiae*), protozoa (*Toxoplasma gondii*), plants (*Solanum lycopersicum*, *Taxus × media* and *Oryza sativa*) and mammals (*Rattus norvegicus*, *Homo sapiens* and *Mus musculus*) [7, 13–18]. Emerging evidence shows that lysine succinylation is involved in metabolism regulation, especially that of glycolysis, the citrate cycle (TCA) and carbohydrate metabolism [19]. In different organisms, most TCA cycle-related enzymes are succinylated [7, 16]. For example, IDH1, a key enzyme that catalyzes the rate-limiting step of the TCA cycle, was identified as a succinylated protein in both microbes and mammals, indicating a potential conserved function for succinylation in the regulation of the TCA cycle [7, 18, 20].

*Dendrobium*, one of the largest genera of the *Orchidaceae*, is widely spread throughout tropical and subtropical Asia and eastern Australia [21, 22]. *Dendrobium officinale*, a critically endangered medicinal herb in the wild, has been used as folk medicine for hundreds of years in China [23, 24]. So far, several chemical components, such as polysaccharides and alkaloids, have been identified in *D. officinale* [25]. Due to their strong immune activities, dendrobium polysaccharides have gained increasing attention and were identified as prominent ingredients in *D. officinale* [26]. In *D. officinale*, the polysaccharide fractions are composed of various monosaccharides, including glucose, mannose and xylose, which are the fundamental building blocks for glycolytic flux [27]. The key enzymes involved in polysaccharide synthesis and metabolic pathways have been partially elucidated in *D. officinale* [28, 29]. Besides, SUMOylation is an important PTM of proteins that involves the reversible conjugation of a small ubiquitin-related modifier polypeptide to substrates [30]. DnSIZ1 protein, a functional homolog of the *Arabidopsis* SIZ1 with SUMO E3 ligase activity, has been identified from *Dendrobium* [31]. Recently, several dendrobine alkaloid associated enzymes, such as cytochrome P450, aminotransferase and methyltransferase, also have been identified in *dendrobium nobile*, suggesting that post-modification enzymes might play vital roles in the biogenetic pathway of dendrobine alkaloid [32]. However, the PTMs of the polysaccharide metabolism-related enzymes in *D. officinale* are largely unknown. The systematic identification of the lysine succinylome of *D. officinale* may aid us in further understanding the biosynthetic pathways of polysaccharides and the molecular basis for the higher polysaccharide content in *D. officinale*.

## Materials and methods

### Plant materials

*D. officinale* seedlings were cultivated in the tissue culture room of the Key Laboratory of Medicinal Plant Germplasm Improvement and Quality Control Techniques in Hangzhou Normal University, Hangzhou, China. Six-month-old tissue culture seedlings were transferred into pots (12 cm diameter) containing a mixture of 300 ml bark, small pebbles and coarse humus soil 3:1:1 (*v*/v/v) at a temperature of 25 ± 1 °C with a light/dark cycle of 12/12 h and 60%–70% relative humidity [33].

### Protein extraction

The *D. officinale* sample was first put in liquid nitrogen and sonicated five times on ice using a high intensity ultrasonic processor (type number JY92-IIN, Scientz, Ningbo, China) in lysis buffer (8 M urea, 1% Triton-100, 10 mM DTT and 0.1% Protease Inhibitor Cocktail IV, 3 μM TSA, 50 mM NAM, 2 mM EDTA). Then, the remaining debris was separated and removed by centrifugation at 20,000×*g* at 4 °C for 15 min. Finally, the protein was precipitated with 15% pre-cooled trichloroacetic buffer for 2 h at −20 °C. After centrifugation 20,000×*g* at 4 °C for 10 min, the supernatant was discarded, and the remaining precipitate was washed with pre-cooled acetone five times. The protein was redissolved in buffer (8 M urea, 100 mM NH_4_CO_3_, pH 8.0) for further tests. The protein concentration was determined using a 2-D Quant kit (GE Healthcare, Uppsala, Sweden) according to the manufacturer’s instructions.

### Trypsin digestion

The protein solution was reduced with 10 mM DTT for 1 h at 37 °C and alkylated with 20 mM iodoacetamide for 45 min at 25 °C in the dark. For trypsin digestion, the protein solution was diluted with 100 mM NH_4_HCO_3_ to a urea concentration of less than 2 M. Finally, trypsin (PTM Biolabs, Hangzhou, China) was added to the protein solution at a 1:50 trypsin-to-protein mass ratio for the first overnight digestion and at a 1:100 trypsin-to-protein mass ratio for the second digestion of 4 h.

### HPLC and affinity enrichment

The protein sample was fractionated by high pH reverse-phase HPLC using an Agilent 300 Extend C18 column with the following parameters: 5 μm particles, 4.6 mm ID and 250 mm length. Briefly, the sample was first separated with a gradient of 2% to 60% acetonitrile in 10 mM ammonium bicarbonate (80 min, pH 10), and then they were combined into eight fractions. To enrich the succinylated peptides, tryptic peptides were first dissolved in NETN buffer (100 mM NaCl, 1 mM EDTA, 50 mM Tris–HCl, 0.5% NP-40, pH 8.0) and then incubated with pre-washed antibody beads (PTM Biolabs, Hangzhou, China) with gentle shaking at 4 °C overnight. The antibody beads were washed with NETN buffer three times and with ddH_2_O twice. The bound peptides were eluted from the beads by 0.1% trifluoroacetic acid buffer. The eluted peptides were combined and cleaned with C18 ZipTips (Millipore, Shanghai, China) according to the manufacturer’s instructions.

### LC-MS/MS analysis

For the LC-MS/MS analysis, the peptides were dissolved in 2% acetonitrile with formic acid and were directly loaded on an Acclaim PepMap 100 reversed-phase pre-column (Thermo scientific, Shanghai, China). Peptide separation was carried out using an Acclaim PepMap RSLC reversed-phase analytical column (Thermo Scientific, Shanghai, China). The LC-MS/MS analysis was performed following the procedure described by our previous publication [18].

### Database search

The resulting MS/MS data was identified using MaxQuant with the integrated Andromeda search engine (version 1.4.1.2). Tandem mass spectra were queried against the *D. officinale* genome, and the transcriptome data were concatenated using the reverse decoy database [29, 34, 35]. Trypsin/P was specified as the cleavage enzyme, allowing up to four missing cleavage sites. The mass error was set to 5 ppm for searching precursor ions and 0.02 Da for searching fragment ions. Succinylation on the N-terminal of an identified protein was specified as a variable modification. False discovery rate thresholds for the modification sites on peptides were specified at 1%. The minimum length of the peptide was set at seven amino acid residues, and the site localization probability was set as >0.75. All of the other parameters used in MaxQuant were set to the defaults.

### Protein annotation methods

The gene ontology (GO)-based annotation of the proteome was obtained from the UniProt-GOA database (http://www.ebi.ac.uk/GOA/). Firstly, the IDs of our identified proteins were converted to UniProt IDs, which could be mapped to the UniProt-GOA database. The rest of the proteins, which were not annotated by the UniProt-GOA database, were annotated by InterProScan software using the protein sequence alignment method. Then, all of the GO annotated proteins were classified into three categories, biological process, cellular component and molecular function. The Kyoto Encyclopedia of Genes and Genomes (KEGG) database was used for the protein pathway annotation. Firstly, the KEGG online service tool ‘KAAS’ was used to annotate the identified protein’s KEGG description. Then, the annotated proteins were mapped on the KEGG pathway using the KEGG online service tool ‘KEGG mapper’. Protein domain functional descriptions were annotated by a sequence analysis application, ‘InterProScan’, using the sequence alignment method. InterPro (http://www.ebi.ac.uk/interpro/) is a database that integrates diverse information, such as protein domains, protein families and protein functional sites. Domains vary in length from between ~25 amino acids up to ~500 amino acids in length. For subcellular localization predictions, the software ‘wolfpsort’ (http://psort.hgc.jp/) was used.

### Motif and motif logo-based clustering analyses

The software ‘motif-x’ (http://motif-x.med.harvard.edu/) was used to analyze the models of specific amino acid sequence sites, 10 amino acids upstream and downstream of the site, in the modify-21-mers in the identified protein sequences. All of the protein sequences in the database were used as a background parameter. The ‘motif score’ was calculated by taking the sum of the negative log probabilities used to fix each site of a given motif. The detailed parameters used for motif identification were the same as previously published [18].

All of the succinylation substrate-enriched categories were collated using their *p* values, and then filtered with *p* value <0.05. The filtered *p* value matrix was transformed by the formula N = −log10 (*p* value). Then, these N values were z-transformed for each motif. These z scores were clustered by one-way hierarchical clustering (Euclidean distance, average linkage clustering) in Genesis. Cluster membership was visualized by a heat map using the “heatmap.2” function from the “gplots” R-package.

### Functional enrichment analysis

For each GO category, a two-tailed Fisher’s exact test was employed to calculate the enrichment of all of the identified protein against the GO database. A correction for multiple hypothesis testing was performed using the standard false discovery rate control method. GO categories with corrected *p* values <0.05 were treated as significant. For each KEGG pathway, a two-tailed Fisher’s exact test was employed to calculate the enrichment of all of the identified proteins against the KEGG database. A correction for multiple hypothesis testing was performed using the standard false discovery rate control method. The KEGG pathway with a corrected *p* value <0.05 is treated as significant. For each protein domain, a two-tailed Fisher’s exact test was employed to calculate the enrichment of all of the identified domains against the protein database. A correction for multiple hypothesis testing was performed using the standard false discovery rate control method. The domain with a corrected *p* value <0.05 was treated as significant. For the bioinformatics analyses, such as the GO-base, KEGG-base and domain-base enrichment, all of the sequences in the database were used as the background [36].

### Protein-protein interaction (PPI) network analysis

All of the identified succinylated peptides were queried against the STRING database v 9.1 (http://string-db.org/) to identify protein-protein interactions. STRING defines a metric named ‘confidence score’ to calculate interaction confidence. All of the interactions with a high confidence score > 0.7 were used to construct the interaction network. Molecular complex detection, a part of the plug-in tool kit of the network analysis, was used to analyze densely connected regions. Cytoscape software (http://www.cytoscape.org/) was used to visualize the interaction network.

### Multiple sequence alignment and phylogenetic tree building

Multiple sequence alignments were performed on the full-length protein sequences in various species using ClustalW (http://www.ebi.ac.uk/Tools/msa/clustalw2/) with the default parameters. The sequence of fructose-bisphosphate aldolase (FBA) from Rabbit Muscle (1ZAH_A) was downloaded from NCBI (http://www.ncbi.nlm.nih.gov/). The alignments were visualized subsequently by GeneDoc software (http://www.nrbsc.org/gfx/genedoc/), and phylogenetic trees related to each glycolytic enzyme were constructed with 10 aligned sequences from different species using MEGA5.1 (http://www.megasoftware.net/mega5/mega.html) employing the neighbor-joining method. Bootstrap values were calculated from 1000 iterations [37]. The sequences of all of the proteins used in our study were obtained from the NCBI protein database.

## Results

### Proteome-wide analysis of lysine succinylation sites on the proteins of *D. officinale*

**Fig. 1:**
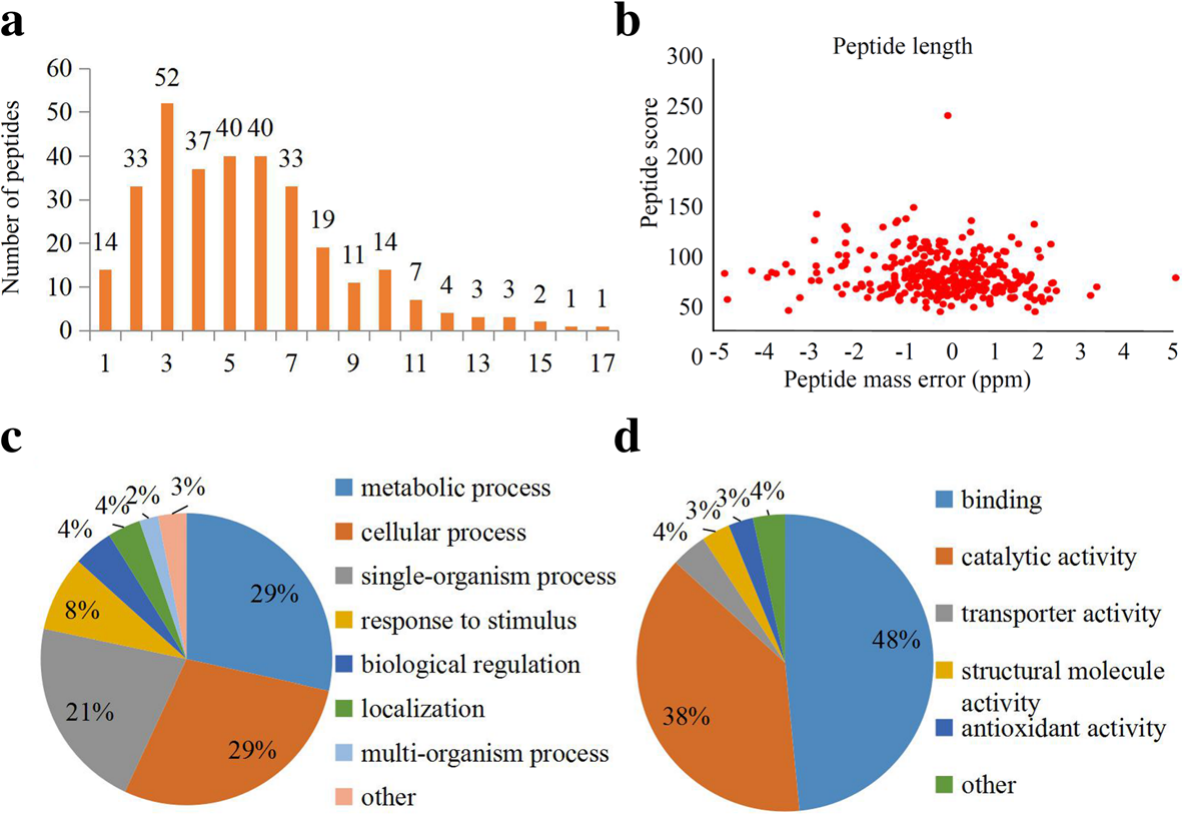
Experimental strategy and the basic information of LC-MS/MS data. **a** The distribution of mass error. **b** T-distribution of succinylated peptides based on their length. GO classification for lysine succinylated proteins, including (**c**) ‘Biological Process’ and (**d**) ‘Molecular Function’

In our study, the lysine-succinylated peptide enrichment, performed with highly sensitive MS and bioinformatics tools, was used to reveal the systemic lysine-succinylated sites and proteins in *D. officinale*. Altogether, 314 lysine succinylation sites in 207 proteins were identified. The mass error of all of the identified peptides is near zero (< 0.02 Da), indicating a high mass accuracy of the MS data (Fig. 1a). In addition, the lengths of most of the identified peptides were distributed from 8 to 18 amino-acid residues, which means the sample preparation method and MS data met the standards (Fig. 1b).

The obtained LC-MS/MS data were used as query against the *D. officinale* genome and transcriptomes (84,299 sequences), and 314 lysine succinylation sites, having scores greater than 40, were identified in our study (Additional file 1). These sites occurred on 207 proteins, which displayed varying abundances, depending on their lengths. The peptides contained different numbers of succinylated sites, ranging from 1 to 11. Out of the 207 succinylated proteins, about 70% (147/207) possessed a single succinylated site, 19.3% (40/207) contained two succinylated sites, 5.3% (11/207) had three succinylated sites, and 4.3% (9/207) possessed more than three succinylated sites. Notably, two proteins, an adenine nucleotide translocator and a succinyl-CoA synthetase beta subunit, had the most extensively succinylated sites (more than eight independent lysine residues) (Additional file 2).

### Functional annotation and subcellular localization analysis of the lysine succinylome in *D. officinale*

A GO analysis is a major bioinformatics initiative to unify the representation of genes or proteins across different species [38]. To understand the possible roles of lysine succinylation in *D. officinale*, GO functional classifications of all of the identified succinylated proteins were performed by searching the UniProt-GOA database. The classification results showed that succinylation occurred on diverse proteins involved in biological processes, cellular components and molecular functions. In the GO term of biological process, most of the succinylated proteins were classed into ‘metabolic process’ (29%) and ‘cellular process’ (29%) (Fig. 1c). In the GO term of cellular component, the largest category of succinylated proteins was ‘cell’ (38%) and the second largest categorize of succinylated proteins was ‘organelle’ (30%). In the GO term of molecular function, the largest category of succinylated proteins consisted of ‘binding’ proteins (48%) and the second largest category, accounting for 38%, consisted of ‘catalytic activity’ proteins (Fig. 1d).

**Fig. 2:**
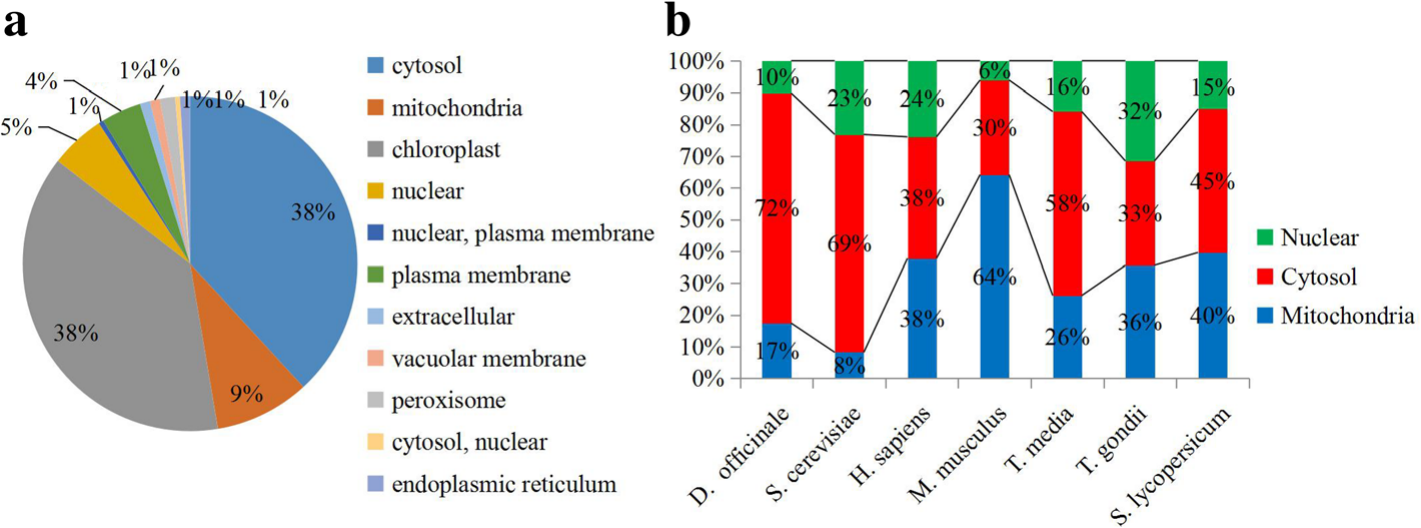
Analysis of subcellular location of lysine succinylated proteins. **a** Subcellular location of lysine succinylated proteins in *D. officinale*. **b** The proportion of succinylated proteins in mitochondria, cytoplasm and nucleus in various organisms

The subcellular localization of a given protein is useful information when predicting its biological function. Therefore, the subcellular localizations of succinylated proteins were analyzed in *D. officinale*. In detail, 38% of the succinylated proteins are located in the cytosol, another 38% of the succinylated proteins are located in the cytosol, 9% of the succinylated proteins are mitochondria-located and 7% of the succinylated proteins are nucleus-located (Fig. 2a). In mammals and bacteria, most of the protein succinylation occurred in the mitochondria, cytoplasm and nucleus [7, 8]. Discarding the chloroplast-located proteins, which mainly exist in plants, the relative proportions of succinylated proteins in three common organelles were calculated and compared among various organisms. The data showed that *D. officinale* possessed the highest proportion of cytosol-located succinylated proteins (72%), twice that in some organisms, including *H. sapiens*, *M. musculus* and *T. gondii* (Fig. 2b).

### Enrichment analysis of the lysine succinylome in *D. officinale*

**Fig. 3:**
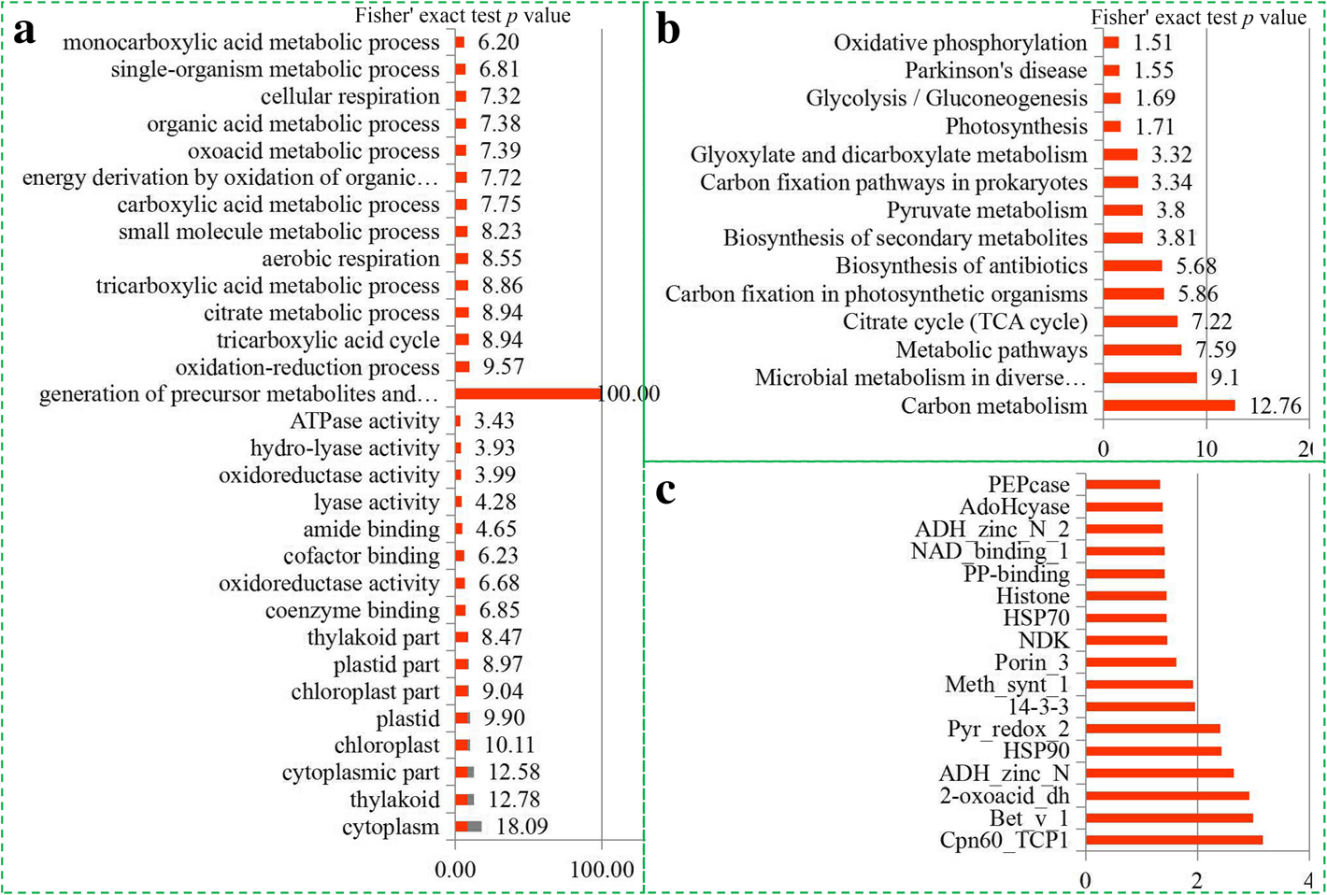
Enrichment analysis of succinylated proteins based on their annotation. **a** GO enrichment analysis of succinylated proteins in *D. officinale*. **b** KEGG enrichment analysis of succinylated proteins in *D. officinale*. **c** Domain enrichment analysis of succinylated proteins in *D. officinale*

To determine the preferred protein types for lysine succinylation, we evaluated the GO enrichment of the succinylated proteins. In biological process, the succinylated proteins related to ‘generation of precursor metabolites and energy’, ‘oxidation-reduction process’ and ‘TCA cycle’ were the most significantly enriched. Meanwhile, significant enrichments of succinylated proteins involved in ‘cytoplasm’, ‘thylakoid’ and ‘cytoplasmic part’ were observed in the cellular component. A wide range of molecular functions related to ‘coenzyme binding’, ‘oxidoreductase activity’ and ‘cofactor binding’ were identified to be significantly enriched with succinylated proteins (Fig. 3a). To obtain more detailed information on the metabolic pathways involved in succinylation, a KEGG enrichment analysis was performed. In total, 38 significantly enriched KEGG pathways were identified. Several metabolic pathways, including ‘carbon metabolism’, ‘TCA cycle’, and ‘carbon fixation in photosynthetic organisms’, were highly enriched in the succinylome of *D. officinale* (Fig. 3b). A protein domain analysis revealed that succinylated proteins in *D. officinale* were mostly highly enriched in Cpn60_TCP1, Bet_v1 and 2-oxoacid_dh domains (Fig. 3c).

### Motifs analysis in identified lysine-succinylated peptides

**Fig. 4:**
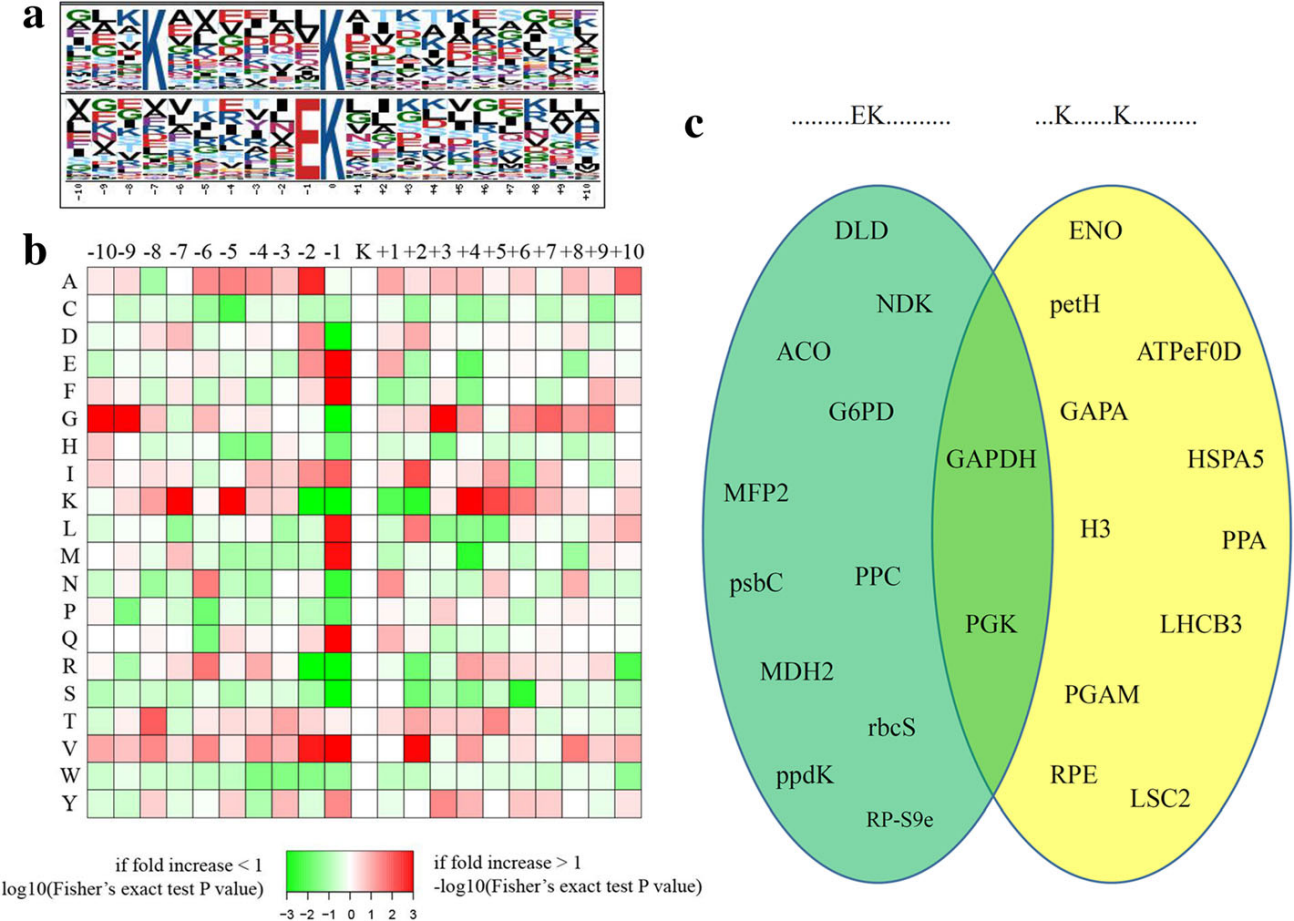
Bioinformatic analysis of lysine succinylation sites in *D. officinale*. **a** Plot shows relative abundance of amino acids flanking succinylated lysine. The relative abundance was counted and schematically represented by an intensity map. The intensity map shows enrichment of amino acids in specific positions of succinylated lysine (10 amino acids upstream and downstream of the succinylation site). **b** Probability sequence motifs of succinylation sites consisting of 10 residues surrounding the targeted lysine residue using Motif-X. Two significantly enriched succinylation site motifs were identified. **c** Analysis of functional preference for different motifs in *D. officinale*. The results were showed by a Venn Diagram

Based on previous studies, diverse succinyl-peptide patterns are present in different organisms [13, 16, 18]. To determine the specific amino acids adjacent to succinylated peptides in *D. officinale*, we counted the amino acid sequences flanking succinylation sites. Two preferred sequence patterns, ***K^suc^******K** (Motif I) and ****EK^suc^*** (Motif II) (* indicates a random amino acid residue and K^suc^ indicates succinylated-K), were identified as conserved succinylation site motifs (Additional file 3). In addition to the succinylated-K, another K was overrepresented in the seventh position upstream of Ksuc sites in Motif I. A strong bias for a glutamic acid (E) upstream of the Ksuc site has also been identified in Motif II (Fig. 4a).

Furthermore, a logo reflecting the relative frequency of amino acids in specific positions of succinyl-21-mers (10 amino acids upstream and downstream of the given site) compared with that of nonsuccinyl-21-mers was constructed to reflect whether there was a significant frequency of specific amino acids surrounding the succinylated lysine site. Our data showed that E had the highest frequency in position −1; while K had the highest frequency in position −7 and the lowest frequency in position −1; and R had highest frequency in position −1 (Fig. 4b). A functional preference of succinylated proteins for different motifs was analyzed in *D. officinale*. For most succinylated proteins, only one motif was contained. Interestingly, both motifs have been identified in glyceraldehyde 3-phosphate dehydrogenase (GAPDH) and phosphoglycerate kinase (PGK) (Fig. 4c and Additional file 4).

### Lysine succinylation on various key enzymes in the glycolysis pathway

Increasing evidence shows that many succinylated proteins were involved in multiple metabolic pathways, including photosynthesis and secondary metabolite biosynthetic pathways [14, 16, 18]. In our study, five key glycolytic enzymes were identified as succinylated proteins, FBA, GAPDH, PGK, phosphoglycerate mutase (PGPG) and enolase (ENO). All of them are also succinylated in different species, including bacteria, mammals and plants, suggesting the potential conserved function of succinylation in the regulation of glycolytic metabolism.

**Fig. 5:**
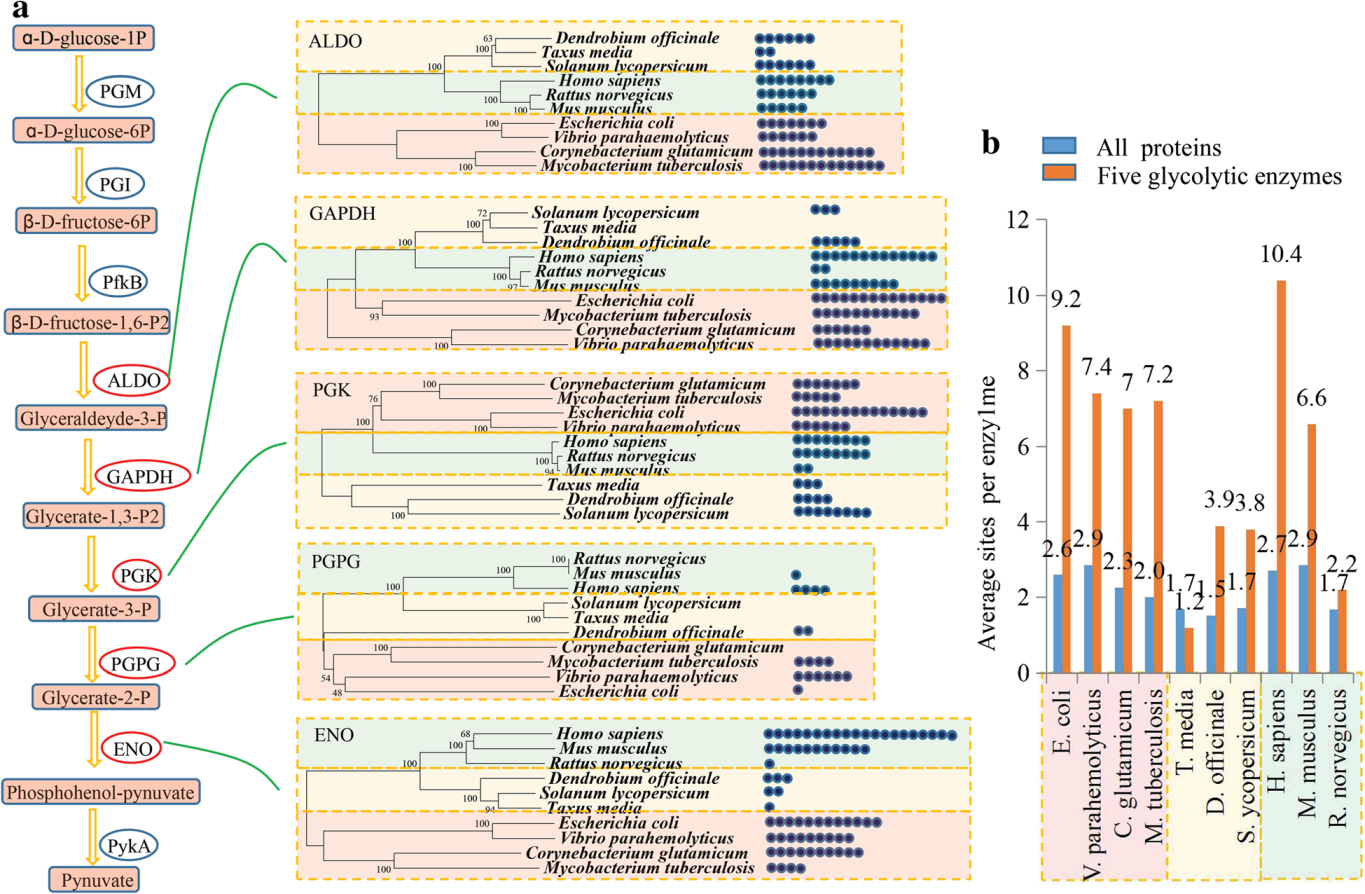
Succinylated enzymes were involved in glycolysis pathway. **a** Succinylated enzymes were involved in glycolysis pathway in *D. officinale*. The red cycles indicate succinylated proteins and *gray cycles* indicate non-succinylated proteins. **b** Five evolutionary trees were built to show the relationships of FBA, GAPDH, PGK, PGPG and ENO among various organisms. The succinylation sites in the five glycolytic enzymes were showed by *blue dots*. **c** The average succinylation sites in one succinylated protein of various organisms

**Fig. 6:**
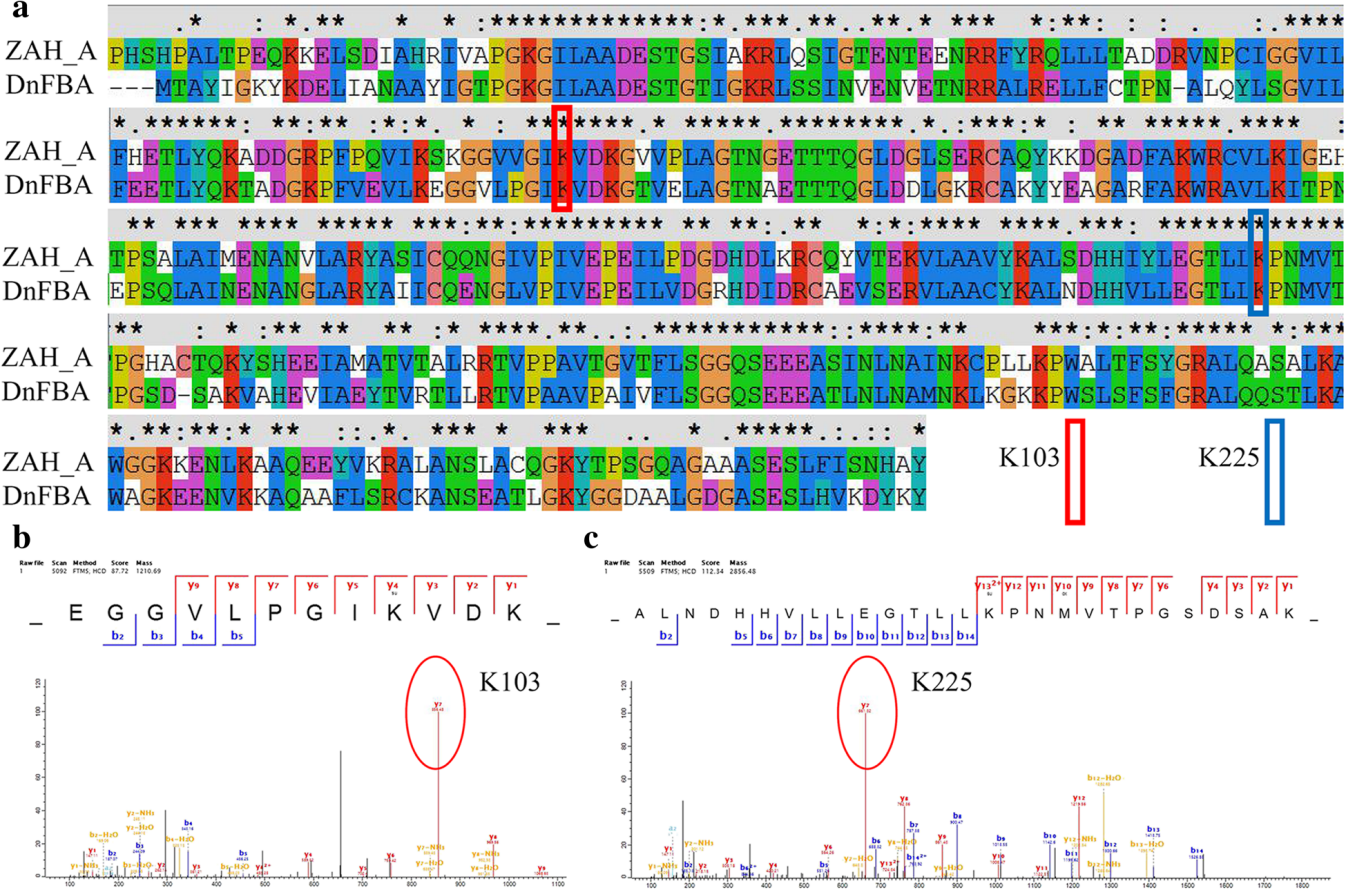
The relationship of active site amino acids residues and lysine succinylation in FBA. **a** Pair-Wise Multiple Sequence Alignment of *D. officinale* and template 1ZAH_A (from Rabbit Muscle). *Red boxes* indicate the succinylated active site lysine residues. **b** Two LC-MS/MS spectra of succinyl-peptides that contained active site lysine residues are shown. *Red cycles* indicate the succinylated active site lysine residues

Based on previous studies, the succinyl-proteome profiling of four bacteria, *E. coli*, *Vibrio parahemolyticus*, *Mycobacterium tuberculosis* and *Corynebacterium glutamicum*, three mammals, *H. sapiens*, *M. musculus* and *R. norvegicus*, and three plants, *T. media*, *D. officinale* and *S. lycopersicum*, have been identified by different groups [7, 14, 16, 17, 39]. In *D. officinale*, six succinylation sites in the FBA, six in GAPDH, six in the PGK, two in PGPG and four in ENO were identified. The number of succinylation sites in the five key glycolytic enzymes of the nine representative species were counted and shown in Fig. 5a. The average number of succinylation sites in one succinylated protein among the various species varied from 1.5 to 2.9. However, in most species, the average numbers of succinylation sites in the five key glycolytic enzymes were greater than the numbers obtained from all of the other succinylated protein. Interestingly, the average succinylation sites in the five enzymes in four bacteria was over seven, and the numbers of average succinylation sites in the five enzymes in three plants were under four (Fig. 6b). The number of succinylation sites in plants was lower than that in bacteria.

The active site’s amino acid residues in the FBA from rabbit muscle (1ZAH A) have been identified by X-ray diffraction with 1.80 Å resolution [40]. Furthermore, we analyzed the active site’s amino acid residues in the FBA of *D. officinale* by a pair-wise multiple sequence alignment with template 1ZAH_A, and the FBA of rabbit was downloaded from NCBI (Fig. 6a). The data showed that two important amino acid residues in the active site could be succinylated (Fig. 6b).

### PPI network of *D. officinale* succinylation substrates

**Fig. 7:**
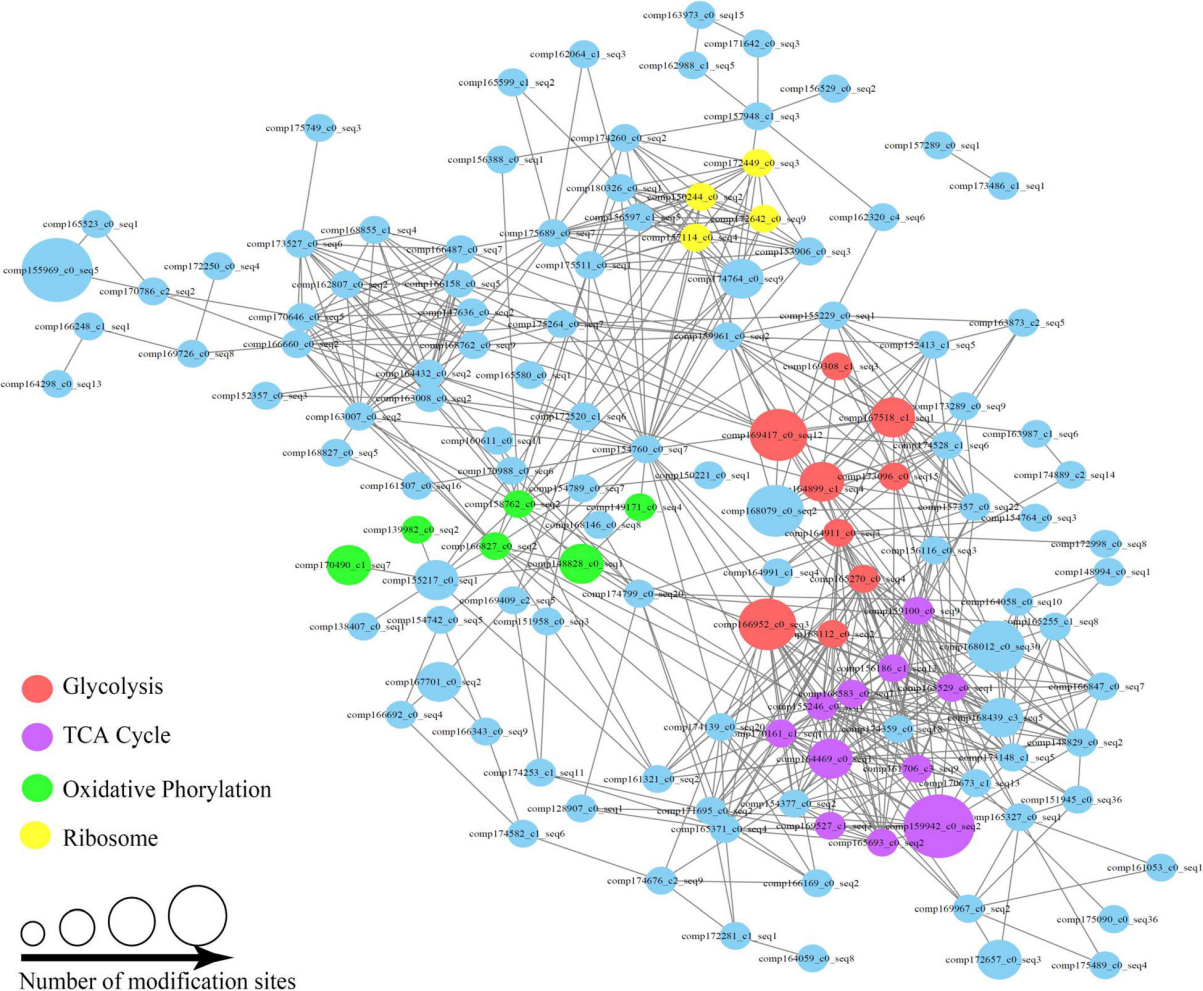
Interaction network of lysine-succinylated proteins in *D. officinale*. The lysine-succinylated proteins in the top four clusters are shown in *red, light purple, cyan* and *yellow*, respectively. *Red* indicates cluster ‘Glycolysis’, light purple indicates cluster ‘TCA Cycle’, cyan indicates cluster ‘Oxidative Phosphorylation’, *yellow* indicates cluster ‘Ribosome’ and *blue* indicates other lysine-succinylated proteins in *D. officinale*

The PPI network analysis can uncover the relationships between different succinylated proteins and the putative biological functions of some unknown proteins. In our study, the PPI network for all succinylated proteins in *D. officinale* was examined using Cytoscape software. The *D. officinale* PPI network consisted of 133 succinylated proteins as nodes, linked by a number of identified direct physical interactions obtained from the STRING database (Additional file 5). A high quality image, as an overview of the PPIs of succinylated proteins in *D. officinale*, was constructed and shown in Fig. 7. The proteins having the functional terms ‘glycolysis’, ‘TCA cycle’, ‘oxidative phosphorylation’ and ‘ribosome’ are highlighted in different colors. For ‘glycolysis’, several well-studied enzymes, such as fructose-bisphosphate aldolase, aldehyde dehydrogenase (ALDH), phosphoglycerate mutase, phosphoglycerate kinase, glyceraldehyde 3-phosphate dehydrogenase, dihydrolipoamide acetyltransferase and enolase, have been identified. For ‘TCA cycle’, many enzymes, including succinyl-CoA synthetases subunits, malate dehydrogenase, 2-oxoglutarate dehydrogenase E1 component, fumarate hydratase, aconitate hydratase 1, isocitrate dehydrogenase and citrate synthase, have been included. Furthermore, a few enzymes assigned to ‘oxidative phosphorylation’, such as inorganic pyrophosphatase, NADH dehydrogenase, F-type H + −transporting ATPase and V-type H + −transporting ATPase, and enzymes related to ‘ribosome’, such as large subunit ribosomal protein and small subunit ribosomal protein, were also identified in PPI networks.

## Discussion

Dendrobium, a large genus in Orchidaceae, is well-known in East Asia due to its high economic and medicinal values [35, 41–43]. Several transcriptomes of *D. officinale* have been sequenced to validate genes related to alkaloid and polysaccharide biosynthesis [29, 44, 45]. However, limited information on the enzymes associated with secondary metabolism has been revealed in *D. officinale*. PTMs are chemical modifications that are evolutionarily conserved, sometimes dynamic, and even reversible in various eukaryotic and prokaryotic cells [8]. Lysine succinylation is a newly reported PTM that activates a reaction intermediate during the transfer of a succinyl unit from succinyl-CoA to homoserine [46].

Combining high-resolution LC-MS/MS and antibody-based affinity enrichment, we identified 314 lysine succinylation sites in 207 *D. officinale* proteins. In *E. coli*, the number of succinylated proteins is 990, which is almost four times more than the number in *D. officinale* [7]. In addition to *E. coli*, two other bacteria (*V. parahemolyticus* and *M. tuberculosis*) and two mammals (*H. sapiens* and *M. musculus*) also contain a large number of succinylated proteins [7, 14, 16, 39]. Interestingly, the number of succinylated proteins in *D. officinale* is similar to that in other identified plant species, such as *T. media* (193 proteins), *S. lycopersicum* (202 proteins) and *O. sativa* (261 proteins). In addition to the number of succinylated proteins, the average succinylation sites in *D. officinale* (3.9 sites per protein) was lower than the average of the published bacteria (more than 7 sites per protein), *H. sapiens* (10.4 sites per protein) and *M. musculus* (6.6 sites per protein). It was higher than the average sites identified in two other plant species, *T. media* (1.2 sites per protein) and *O. sativa* (2.5 sites per protein), and similar to that in *S. lycopersicum* (3.8 sites per protein) (Fig. 5b). The data suggested a decrease in the relative frequency of succinylation during the evolution from bacteria to plants, despite most lysine succinylation occurring at low levels in evolutionarily diverse organisms [7]. Certainly, any experimental steps, such as initial enrichment, may affect the final identified numbers. Thus, more further studies were needed to check the number difference between *D. officinale* and the previous published work in other species.

The succinyl-proteome profiling of mammals showed that succinylated proteins mainly exist in the mitochondria, cytoplasm and nucleus [8, 9]. In *H. sapiens* and *M. musculus*, more than half of the succinylation sites occur on mitochondrial proteins, while this was only 9% in *D. officinale* [7]. The MS analysis readily captured the relatively abundant proteins, such as chloroplast proteins in plants. When discarding chloroplast proteins, data still indicated a significant difference in the subcellular distribution of lysine succinylation between *D. officinale* and other identified organisms (Fig. 2b). A large proportion of succinylation sites occurred on cytosol-located proteins, suggesting the involvement of succinylation in *D. officinale* metabolism. Besides, several membrane-located proteins also were identified. For example, membrane-located pyrophosphatases are ubiquitous enzymes that are critical for phosphate metabolism in various biologically important molecules such as proteins, nucleic acids, and fatty acids [47]. Interestingly, three succinylation sites were identified in an pyrophosphatase, suggesting a possible role of succinylation in this catalytic process in *D. officinale*. Moreover, mitochondrial carrier family proteins consist of six membrane spanning helices and catalyze the specific transport of various substrates [48]. A mitochondrial carrier with eight succinylation sites was identified in *D. officinale*. Then, a 14–3-3 like protein was identified as plasma membrane-located protein in *D. officinale* [49], indicating a diversified cellular role of succinylation.

In mammals, the sequence logos do not reveal a significant bias for a particular amino acid; however, in several bacteria and plants, many specific motifs have been uncovered [7]. In *D. officinale*, two preferred sequence patterns, ^***^Ksuc^******^K^**^ (Motif I) and ^****^EKsuc^***^ (Motif II), were identified (Fig. 4a). Interestingly, the Motif I in *D. officinale* had already been reported in rice and *M. tuberculosis*, and the Motif II in *D. officinale* had already been reported in *V. parahemolyticus* and tomato [14, 17, 50]. This suggested that several motifs may be shared by both plants and bacteria. Furthermore, Pan’s group reported that succinylated proteins with different functions have significant preferences for specific motifs [14]. In *D. officinale*, no significant functional preference for motifs has been found. For example, both two motifs have been identified in GAPDH and PGK, which are two key enzymes in the glycolysis pathway. In the TCA cycle, LSC2 prefers Motif I and two other enzymes, ACO and MDH2, prefer Motif II (Fig. 4c).

Recent studies revealed that polysaccharides are major active ingredients in *D. officinale* [51]. In our study, we focused on the enzymes involved in the glycolysis pathway, which is the metabolic pathway upstream of polysaccharide biosynthesis. In *D. officinale*, succinylation occurred on five key enzymes, and the average number of sites in each glycolysis-related enzyme is 3.9, which is significantly higher than the average number of sites in each glycolysis-unrelated protein (about 1.5). In addition, the subnetwork of glycolysis showed a relatively high enrichment in *D. officinale*, and the results confirmed the involvement of succinylation in the regulation of the glycolysis pathway. The succinylation of glycolytic enzymes widely exists in microbes and mammals [3, 7, 16]. According to the phylogenetic trees, most sequences, related to a given glycolytic enzyme, showed a close relationship (bootstrap value >95) among different organisms. The evolutionary convergence in protein sequences of glycolytic enzymes from diverse organisms may explain the ubiquity of the succinylation of glycolytic enzymes (Fig. 5a). The density of lysine succinylation sites, nevertheless, was significantly different among organisms, suggesting that succinylation may be involved in the regulation of glycolytic flux in various ways (Fig. 5b).

Furthermore, in *D. officinale*, six succinylation sites have been identified in FBA, which is a key enzyme of the glycolytic pathway and responsible for the reversible cleavage of fructose 1,6-bisphosphate to glyceraldehyde 3-phosphate and dihydroxyacetone phosphate [52]. In our study, the FBA active site’s amino acids residues from *D. officinale* were identified. Interestingly, two of these active site residues, K103 and K225, could be succinylated, indicating a potential function of lysine succinylation in the regulation of glycolytic enzyme activities (Fig. 6).

Glycolysis, oxidization of glucose to pyruvate, is a central metabolic pathway. The PPI network confirmed that the glycolytic enzymes showed relatively high enrichment in *D. officinale*. Except for FBA, ALDH is another important enzyme that oxidize a wide range of endogenous and exogenous aliphatic and aromatic aldehydes [53]. In *Arabidopsis*, *ALDH* genes play roles in stress responses, indicating that succinylated-ALDH may participate in several stress-associated pathways in *D. officinale* [54]. Besides, phosphoglycerate mutase, a enzyme involved in the reversible interconversion of 3-phosphoglycerate to 2-phosphoglycerate, is required for normal pollen development in *Arabidopsis* [55]. Our data suggested that succinylated-phosphoglycerate mutase may be critical for fertility in *D. officinale*.

## Conclusion

We presented a comprehensive large-scale succinyl-proteome of *D. officinale*, a traditional medicinal herb. In total, 314 lysine succinylation sites, occurring on 207 proteins, were identified in *D. officinale*. A large number of succinylated proteins involved in a broad spectrum of functions, ranging from protein binding to molecule activity, indicated a vital role in the regulation of the physiology and biochemical processes in *D. officinale*. Moreover, our data provides a basic resource for the functional validation of several succinylated proteins in the glycolysis pathway. This may assist us in understanding the molecular basis of polysaccharide synthesis in *D. officinale*.

## Additional files

Additional file 1: The information of succinylated sites in each succinylated protein (XLSX 93 kb)

Additional file 2: Number of modified site in a protein (DOCX 12 kb)

Additional file 3: Information of two Motifs in *D. officinale* (XLSX 11 kb)

Additional file 4: Functional preference of succinylated proteins for different motifs in *D. officinale* (XLSX 13 kb)

Additional file 5: Detail information on identified direct physical interactions obtained from the STRING database (XLSX 70 kb)

## Abbreviations

ACO: Aconitate hydratase
E: glutamic acid
ENO: Enolase
FBA: Fructose-bisphosphate aldolase
GAPDH: Glyceraldehyde 3-phosphate dehydrogenase
GO: Gene ontology
HPLC: High Performance Liquid Chromatography
IDH1: Isocitrate dehydrogenase 1 (NADP+)
KEGG: Kyoto Encyclopedia of Genes and Genomes
LC-MS/MS: liquid chromatography–tandem mass spectrometry
LSC: Succinyl-CoA ligase
MDH2: Malate dehydrogenase
PGK: Phosphoglycerate kinase
PGPG: Phosphoglycerate mutase
PPI: Protein-protein interaction
PTM: Post-translational modification
TCA: citrate cycle

## References

[1] Walsh CT, Garneau-Tsodikova S, Gatto GJ (2006). Protein posttranslational modifications: the chemistry of proteome diversifications. Angew Chem Int Ed.

[2] Huang H, Lin S, Garcia BA, Zhao Y (2015). Quantitative proteomic analysis of histone modifications. Chem Rev.

[3] Peng C, Lu Z, Xie Z, Cheng Z, Chen Y, Tan M et al. The first identification of lysine malonylation substrates and its regulatory enzyme. Molecular & Cellular Proteomics:MCP. 2011; 10(12):M111 012658. 10.1074/mcp.M111.012658 PMC3237090 21908771

[4] Hershko A, Ciechanover A (1998). The ubiquitin system. Annu Rev Biochem.

[5] Choudhary C, Kumar C, Gnad F, Nielsen ML, Rehman M, Walther TC (2009). Lysine acetylation targets protein complexes and co-regulates major cellular functions. Science.

[6] Xiao H, Smeekens JM, Wu R (2016). Quantification of tunicamycin-induced protein expression and N-glycosylation changes in yeast. Analyst.

[7] Weinert BT, Schölz C, Wagner SA, Iesmantavicius V, Su D, Daniel JA (2013). Lysine succinylation is a frequently occurring modification in prokaryotes and eukaryotes and extensively overlaps with acetylation. Cell Rep.

[8] Xie Z, Dai J, Dai L, Tan M, Cheng Z, Wu Y (2012). Lysine succinylation and lysine malonylation in histones. Molecular & Cellular Proteomics: MCP.

[9] Zhang Z, Tan M, Xie Z, Dai L, Chen Y, Zhao Y (2011). Identification of lysine succinylation as a new post-translational modification. Nat Chem Biol.

[10] Ran R, Becher D, Büttner K, Biran D, Hecker M, Ron EZ (2004). Probing the active site of homoserine Trans -succinylase. FEBS Lett.

[11] Hasan MM, Yang S, Zhou Y, Mollah MN (2016). SuccinSite: a computational tool for the prediction of protein succinylation sites by exploiting the amino acid patterns and properties. Mol BioSyst.

[12] Du J, Zhou Y, Su X, Yu JJ, Khan S, Jiang H (2011). Sirt5 is a NAD-dependent protein lysine demalonylase and desuccinylase. Science.

[13] Li X, Hu X, Wan Y, Xie G, Li X, Chen D (2014). Systematic identification of the lysine succinylation in the protozoan parasite *Toxoplasma gondii*. J Proteome Res.

[14] Pan J, Chen R, Li C, Li W, Ye Z (2015). Global analysis of protein lysine succinylation profiles and their overlap with lysine acetylation in the marine bacterium *Vibrio parahemolyticus*. J Proteome Res.

[15] Cheng Y, Hou T, Ping J, Chen G, Chen J (2016). Quantitative succinylome analysis in the liver of non-alcoholic fatty liver disease rat model. Proteome Sci.

[16] Xie L, Liu W, Li Q, Chen S, Xu M, Huang Q (2015). First succinyl-proteome profiling of extensively drug-resistant *Mycobacterium tuberculosis* revealed involvement of succinylation in cellular physiology. J Proteome Res.

[17] Jin W, Wu F (2016). Proteome-wide identification of lysine succinylation in the proteins of tomato (*Solanum lycopersicum*). PLoS One.

[18] Shen C, Xue J, Sun T, Guo H, Zhang L, Meng Y (2016). Succinyl-proteome profiling of a high taxol containing hybrid *Taxus* species (*Taxus x media*) revealed involvement of succinylation in multiple metabolic pathways. Sci Rep.

[19] Colak G, Xie Z, Zhu AY, Dai L, Lu Z (2013). Zhang yet al. Identification of lysine succinylation substrates and the succinylation regulatory enzyme CobB in *Escherichia coli*. Mol Cell Proteomics.

[20] Izquierdo-Garcia JL, Cai LM, Chaumeil MM, Eriksson P, Robinson AE, Pieper RO et al. Glioma cells with the IDH1 mutation modulate metabolic fractional flux through pyruvate carboxylase. PLoS One. 2014; 9(9):e108289-e108289. 10.1371/journal.pone.0108289 PMC4171511 25243911

[21] Harriman NA (2012). Flora of china illustrations. Orchidaceae Economic Botany.

[22] Cheng X, Chen W, Zhou Z, Liu J, Wang H (2013). Functional characterization of a novel tropinone reductase-like gene in *Dendrobium nobile* Lindl. J Plant Physiol.

[23] Yang L, Wang Z, Xu L (2006). Simultaneous determination of phenols (bibenzyl, phenanthrene, and fluorenone) in *Dendrobium* species by high-performance liquid chromatography with diode array detection. J Chromatogr A.

[24] Xu X, Wang C, Ma X, Pan Y, Ying Q, Song H (2015). Overexpression of DnWRKY29 in tobacco impaired plants tolerance to salt and drought stresses. Russ J Plant Physiol.

[25] Juan LI, Shunxiang LI, Dan H, Zhao X, Cai G (2011). Advances in the of resources, constituents and pharmacological effects of *Dendrobium officinale*. Science & Technology Review.

[26] Meng LZ, Lv GP, Hu DJ, Cheong KL, Xie J, Zhao J (2013). Effects of polysaccharides from different species of *Dendrobium* (Shihu) on macrophage function. Molecules.

[27] Fan Y, He X, Zhou S, Luo A, He T, Chun Z (2009). Composition analysis and antioxidant activity of polysaccharide from *Dendrobium denneanum*. Int J Biol Macromol.

[28] Zhang J, He C, Wu K (2016). Teixeira da Silva JA. Zeng Set al Transcriptome analysis of Dendrobium officinale and its application to the identification of genes associated with polysaccharide synthesis Frontiers in Plant Science.

[29] Zhang GQ, Xu Q, Bian C, Tsai WC, Yeh CM, Liu KW et al. The *Dendrobium catenatum* Lindl. genome sequence provides insights into polysaccharide synthase, floral development and adaptive evolution. Scientific Reports. 2016; 6:19029. 10.1038/srep19029 PMC4709516 26754549

[30] Geissfriedlander R, Melchior F (2007). Concepts in sumoylation: a decade on. Nat Rev Mol Cell Biol.

[31] Liu F, Wang X, Su M, Yu M, Zhang S, Lai J (2015). Functional characterization of DnSIZ1, a SIZ/PIAS-type SUMO E3 ligase from *Dendrobium*. BMC Plant Biol.

[32] Li Q, Ding G, Li B, Guo SX. Transcriptome analysis of genes involved in dendrobine biosynthesis in *Dendrobium nobile* Lindl. infected with *Mycorrhizal Fungus* MF23 (*Mycena* sp.). Scientific Reports. 2017; 7(1):316. 10.1038/s41598-017-00445-9 PMC5428410 28331229

[33] Wu JB, Zhang CL, Mao PP, Qian YS, Wang HZ. First report of leaf spot caused by Nigrospora oryzae on Dendrobium candidum in China. Plant Dis 2014; 98(7):996–996. 10.1094/PDIS-09-13-1006-PDN 30708927

[34] Yan L, Wang X, Liu H, Tian Y, Lian J, Yang R (2015). The genome of *Dendrobium officinale* illuminates the biology of the important traditional Chinese orchid herb. Mol Plant.

[35] Meng Y, Yu D, Xue J, Lu J, Feng S, Shen C (2016). A transcriptome-wide, organ-specific regulatory map of *Dendrobium officinale*, an important traditional Chinese orchid herb. Sci Rep.

[36] Shen C, Yang Y, Liu K, Zhang L, Guo H, Sun T (2016). Involvement of endogenous salicylic acid in iron-deficiency responses in *Arabidopsis*. J Exp Bot.

[37] Shen C, Yue R, Bai Y, Feng R, Sun T, Wang X (2015). Identification and analysis of *Medicago truncatula* auxin transporter gene families uncover their roles in responses to *Sinorhizobium meliloti* infection. Plant & Cell Physiology.

[38] Young MD, Wakefield MJ, Smyth GK, Oshlack A (2010). Gene ontology analysis for RNA-seq: accounting for selection bias. Genome Biol.

[39] Mizuno Y, Nagano-Shoji M, Kubo S, Kawamura Y, Yoshida A, Kawasaki H (2016). Altered acetylation and succinylation profiles in *Corynebacterium glutamicum* in response to conditions inducing glutamate overproduction. MicrobiologyOpen.

[40] Stjean M, Lafrancevanasse J, Liotard B, Sygusch J (2005). High resolution reaction intermediates of rabbit muscle fructose-1,6-bisphosphate aldolase: substrate cleavage and induced fit. J Biol Chem.

[41] Takamiya T, Wongsawad P, Tajima N, Shioda N, Lu JF, Wen CL (2011). Identification of *Dendrobium* species used for herbal medicines based on ribosomal DNA internal transcribed spacer sequence. Biol Pharm Bull.

[42] Lu JJ, Suo NN, Hu X, Wang S, Liu JJ, Wang HZ (2012). Development and characterization of 110 novel EST-SSR markers for *Dendrobium officinale* (*Orchidaceae*). Am J Bot.

[43] Sun Y, Shen Y, Li A, Fu W (2014). Ectopic expression of *Dendrobium EREB5* gene in *Arabidopsis* influences leaf morphology. In Vitro Cellular & Developmental Biology – Plant.

[44] Guo X, Li Y, Li C, Luo H, Wang L, Qian J (2013). Analysis of the *Dendrobium officinale* transcriptome reveals putative alkaloid biosynthetic genes and genetic markers. Gene.

[45] Shen C, Guo H, Chen H, Shi Y, Meng Y, Lu J (2017). Identification and analysis of genes associated with the synthesis of bioactive constituents in *Dendrobium officinale* using RNA-Seq. Sci Rep.

[46] Rosen R, Becher D, Buttner K, Biran D, Hecker M, Ron EZ (2004). Probing the active site of homoserine trans-succinylase. FEBS Lett.

[47] Kornberg A (1948). The participation of inorganic pyrophosphate in the reversible enzymatic synthesis of diphosphopyridine nucleotide. J Biol Chem.

[48] Haferkamp I, Schmitz-Esser S (2012). The plant mitochondrial carrier family: functional and evolutionary aspects. Front Plant Sci.

[49] Qin C, Cheng L, Shen J, Zhang Y, Cao H, Lu D (2016). Genome-wide identification and expression analysis of the 14-3-3 family genes in *Medicago truncatula*. Front Plant Sci.

[50] He D, Wang Q, Li M, Damaris RN, Yi X, Cheng Z, Yang P (2016). Global proteome analyses of lysine acetylation and succinylation reveal the widespread involvement of both modification in metabolism in the embryo of germinating rice seed. J Proteome Res.

[51] Zhang J, Wu K, Zeng S (2013). Teixeira da Silva JA, Zhao X. Tian CE et al Transcriptome analysis of Cymbidium sinense and its application to the identification of genes associated with floral development BMC Genomics.

[52] Prasad CS, Gupta S, Kumar H, Tiwari M (2012). Evolutionary and functional analysis of fructose bisphosphate aldolase of plant parasitic nematodes. Bioinformation.

[53] Kirch HH, Bartels D, Wei Y, Schnable PS, Wood AJ (2004). The *ALDH* gene superfamily of *Arabidopsis*. Trends Plant Sci.

[54] Kirch HH, Schlingensiepen S, Kotchoni S, Sunkar R, Bartels D (2005). Detailed expression analysis of selected genes of the *aldehyde dehydrogenase* (*ALDH*) gene superfamily in *Arabidopsis thaliana*. Plant Mol Biol.

[55] Zhao Z, Assmann SM (2011). The glycolytic enzyme, phosphoglycerate mutase, has critical roles in stomatal movement, vegetative growth, and pollen production in *Arabidopsis thaliana*. J Exp Bot.

