# Supplementary material for: Succinyl-proteome profiling of Dendrobium officinale, an important traditional Chinese orchid herb, revealed involvement of succinylation in the glycolysis pathway
Source: BMC Genomics. 2017 Aug 10;18:598. doi: 10.1186/s12864-017-3978-x (PMC5553593; doi:10.1186/s12864-017-3978-x)
Supplement: Supplementary file 2 — Number of modified site in a protein (DOCX 12 kb) [file 12864_2017_3978_MOESM2_ESM.docx]

Figure S2 Number of modified site in a protein.
